# The modification of Gat1p in nitrogen catabolite repression to enhance non-preferred nitrogen utilization in *Saccharomyces cerevisiae*

**DOI:** 10.1038/srep21603

**Published:** 2016-02-22

**Authors:** Xinrui Zhao, Huijun Zou, Jian Chen, Guocheng Du, Jingwen Zhou

**Affiliations:** 1 The Key Laboratory of Industrial Biotechnology, Ministry of Education, School of Biotechnology, Jiangnan University, 1800 Lihu Road, Wuxi, Jiangsu, China; 2 Synergetic Innovation Center of Food Safety and Nutrition, 1800 Lihu Road, Wuxi, Jiangsu 214122, China; 3 Zhejiang Guyuelongshan Shaoxing Wine Company, 13 Yangjiang Road, Shaoxing, Zhejiang, China

## Abstract

In *Saccharomyces cerevisiae*, when preferred nitrogen sources are present, the metabolism of non-preferred nitrogen is repressed. Previous work showed that this metabolic regulation is primarily controlled by nitrogen catabolite repression (NCR) related regulators. Among these regulators, two positive regulators (Gln3p and Gat1p) could be phosphorylated and sequestered in the cytoplasm leading to the transcription of non-preferred nitrogen metabolic genes being repressed. The nuclear localization signals (NLSs) and nuclear localization regulatory signals (NLRSs) in Gln3p and Gat1p play essential roles in the regulation of their localization in cells. However, compared with Gln3p, the information of NLS and NLRS for Gat1p remains unknown. In this study, residues 348–375 and 366–510 were identified as the NLS and NLRS of Gat1p firstly. In addition, the modifications of Gat1p (mutations on the NLS and truncation on the NLRS) were attempted to enhance the transcription of non-preferred nitrogen metabolic genes. Quantitative real-time PCR showed that the transcriptional levels of 15 non-preferred nitrogen metabolic genes increased. Furthermore, during the shaking-flask culture tests, the utilization of urea, proline and allantoine was significantly increased. Based on these results, the genetic engineering on Gat1p has a great potential in enhancing non-preferred nitrogen metabolism in *S. cerevisiae*.

Most of microorganisms can sense changes in the amounts and quality of nutrients, allowing their optimal utilization in highly competitive environments^1^. For example, *Saccharomyces cerevisiae* can utilize various kinds of nitrogenous compounds, but with distinct preferences^2^. In the presence of preferred nitrogen sources, transcription of the genes associated with the transport and utilization of non-preferred nitrogen are repressed. This phenomenon is controlled by nitrogen catabolite repression (NCR)^3^. In the regulation of NCR, the key step is the activation of TOR complex 1 (TorC1) leading to the phosphorylation of several activators for non-preferred nitrogen metabolism^4^.

Among these activators, Gln3p and Gat1p are two important global regulators^3^. The transcription of Gln3p is constitutive in cells, but its intracellular distribution is regulated by the type of nitrogen in the environment^5^. The regulation of intracellular distribution of Gat1p is similar to Gln3p, but its transcription requires the activation of Gln3p^6^. In the presence of preferred nitrogen sources, Tor1p and some unknown phosphokinases are activated, leading to the phosphorylation of Gln3p and Gat1p^6^,^7^. After being phosphorylated, Gln3p and Gat1p are excluded by nuclear membrane^8^. Without the activation of Gln3p and Gat1p, the transcription of many genes are repressed, including *DUR1,2* (encoding urea amidolyase), *DUR3* (encoding urea permease), *CAR1* (encoding arginase)^3^.

The phosphorylation of Gln3p and Gat1p is very important for their intracellular distribution^9^, but only the phosphorylation sites in Gln3p have been reported. Its phosphorylation sites were identified as three serines at residues 344, 347 and 355, which all localized in the nuclear localization signal (NLS) in Gln3p^5^. The NLS is a region in eukaryotic regulators, which mediates interactions between a regulator and the nuclear pore complex to control its nuclear entry^10^. When three serines in NLS were mutated to aspartates, intranuclear Gln3p_S344D_, _S347D_, _S355D_ decreased markedly^5^. Whether these serines are phosphorylated is dependent on the other regions in Gln3p, which we call these regions as nuclear localization regulatory signals (NLRS) in this article. These NLRS can combine with other upstream regulators, such as Tor1p, to induce environmentally triggered phosphorylation in Gln3p^11^. Previous research showed that lack of these regions would partially dissociate the interaction between Gln3p and Tor1p^11^. Up to now, only the NLRS of Gln3p (654–667) has been characterized^5^,^11^.

In our previous study, based on the information of NLS and NLRS, we successfully applied different strategies to relieve the effect of NCR on Gln3p^12^. Genetically modified strains could markedly reduce both the concentration of urea and ethyl carbamate in the model rice wine system^12^. Since the transcription of some other non-preferred nitrogen metabolic genes (such as *CAN1* and *PUT2*) are not deeply dependent on Gln3p, it is possible to find even more effective strategies on other regulators^3^. Gat1p, which also shares high homology with Gln3p^13^ and occupies a central position in NCR regulation^6^, is a suitable alternative to perform metabolic engineering on NCR.

However, no NLS sequences and relative phosphorylation sites for Gat1p were available and the information of NLRS for Gat1p has not been reported. Therefore, in this study, the NLS and NLRS of Gat1p were determined and the roles of these regulatory regions were confirmed. Several metabolic engineering strategies were performed on Gat1p to derepress the effect of NCR. The results of qRT-PCR and shaking-flask culture tests showed that NCR effect could be significantly decreased by the modifications on Gat1p. Based on the understanding of NLS and NLRS in Gat1p, more comprehensive and accurate view of the regulation of nitrogen metabolism in *S. cerevisiae* could be obtained and some new strategies on Gat1p could be applied to reduce the concentration of non-preferred nitrogen and potential carcinogenic compounds during fermented foods production.

## Results

### Potential NLS and NLRS in Gat1p

**Figure 1:**
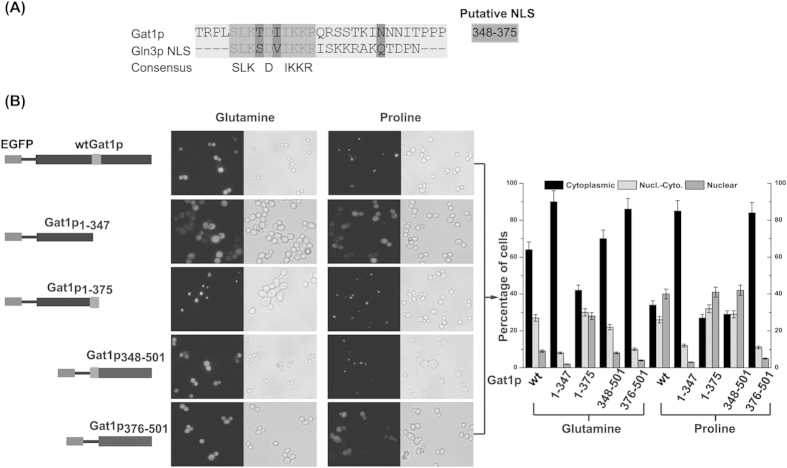
Identification of the putative NLS sequences of Gat1p. (**A**) Sequence alignment of the precise NLS of Gln3p with the Gat1p. (**B**) Left: localization of the EGFP-wtGat1p, EGFP-Gat1p_1–347_, EGFP-Gat1p_1–375_, EGFP-Gat1p_348–501_, and EGFP-Gat1p_376–501_ in glutamine- or proline-grown transformants. Right: the percentage of cells displaying cytoplasmic and nuclear fluorescence for each protein. Black, light gray, and dark gray bars represent proteins located in the cytoplasm only, in both the cytoplasm and nucleus, and in the nucleus only, respectively. Error bars represent the standard deviation of mean of three replicates.

Based on an alignment of the NLS sequences of Gln3p and Gat1p, the potential NLS for Gat1p were identified at residues 348–375 (Fig. 1A). To confirm whether the region is responsible for the nuclear localization, *S. cerevisiae* CEN.PK2-1C was transformed with a series of EGFP-fusion expression vectors encoding partially truncated fragments of Gat1p (Gat1p_1–347_, Gat1p_1–375_, Gat1p_348–510_, and Gat1p_376–510_). The EGFP fused with intact Gat1p (wtGat1p) was used as a control. Compared with the intracellular distribution of wtGat1p, regardless of whether the nitrogen source was preferred (glutamine) or non-preferred (proline), Gat1p_1–347_ and Gat1p_376–510_, which lack the potential NLS, could not be observed in the nucleus (Fig. 1B). This suggests that residues 348–375 in Gat1p play an important role in its localization. Besides, when the C-terminal region of Gat1p was truncated, the nuclear proportion of Gat1p_1–375_ (28%) was much higher than other truncated-Gat1p fragments even when glutamine was present (Fig. 1B). In other words, lack of residues 376–510 derepressed the nuclear exclusive effect on Gat1p. This result indicates that there might be a NLRS in the C-terminal region of Gat1p, which controls the localization of Gat1p between the cytoplasm and the nucleus.

### Potential phosphorylation sites in NLS of Gat1p

**Figure 2:**
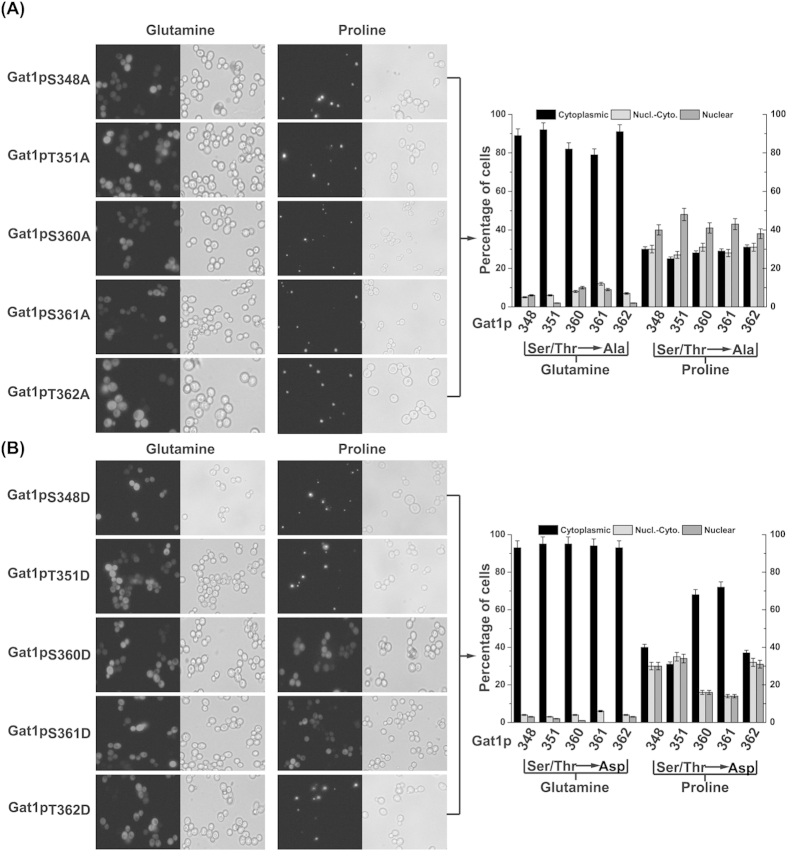
Localization of Gat1p with mutations in NLS. (**A**) Left: localization of EGFP-Gat1p in which the serines and threonines in the NLS were replaced with alanine. Right: the percentage of cells displaying cytoplasmic and nuclear fluorescence for each mutated protein. (**B**) Left: localization of EGFP-Gat1p in which the serines and threonines in the NLS were replaced with aspartate. Right: the percentage of cells displaying cytoplasmic and nuclear fluorescence for each mutated protein. Black, light gray, and dark gray bars represent proteins located in the cytoplasm only, in both the cytoplasm and nucleus, and in the nucleus only, respectively. Error bars represent the standard deviation of mean of three replicates.

Because serine and threonine are the common phosphorylation sites in *S. cerevisiae*^14^, the residues at 348 (serine), 351 (threonine), 360 (serine), 361 (serine), and 362 (threonine) are potential phosphorylation sites in the NLS of Gat1p. To determine whether these residues are required for the nuclear localization of Gat1p, all of them were mutated to alanine or aspartate^5^. Compared with the intracellular distribution of wtGat1p (Gat1p without mutations) (Fig. 1B), when the medium was supplemented with glutamine (preferred nitrogen) as the only nitrogen source, intranuclear Gat1p_S348A_, Gat1p_T351A_, and Gat1p_T362A_ did not increase, whereas intranuclear Gat1p_S360A_ and Gat1p_S361A_ increased slightly (Fig. 2A). When the medium was supplemented with proline (non-preferred nitrogen) as the only nitrogen source, intranuclear Gat1p_S348D_, Gat1p_T351D_, and Gat1p_T362D_ decreased slightly, whereas intranuclear Gat1p_S360D_ and Gat1p_S361D_ decreased markedly (Fig. 2B). These results suggest that the residues at 360 (serine) and 361 (serine) are the phosphorylation sites in the NLS of Gat1p.

### The effect of mutations and truncation on the NLS and NLRS in Gat1p

**Figure 3:**
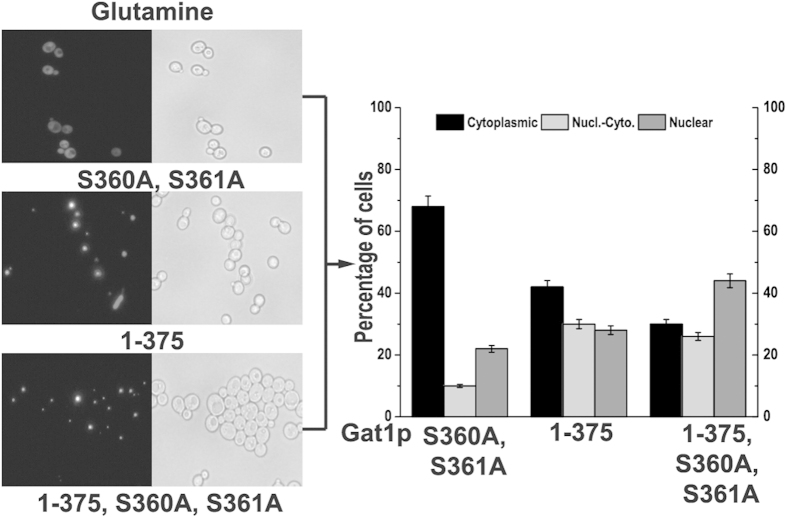
Effects of the modifications of Gat1p on its intracellular distribution. Left: localization of EGFP-Gat1p_S360A, S361A_, EGFP-Gat1p_1–375_, and EGFP-Gat1p_1–375, S360A, S361A_ in glutamine-grown transformants. Right: the percentage of cells displaying cytoplasmic and nuclear fluorescence for each modified protein. Black, light gray, and dark gray bars represent proteins located in the cytoplasm only, in both the cytoplasm and nucleus, and in the nucleus only, respectively. Error bars represent the standard deviation of mean of three replicates.

Based on the NLS and NLRS of Gat1p obtained in this work, several strategies (mutations and truncation) were applied to relieve the effect of NCR. pYX212-EGFP-Gat1p_S360A, S361A_, pYX212-EGFP-Gat1p_1–375_, and pYX212-EGFP-Gat1p_1–375, S360A, S361A_ were constructed and their intracellular distributions were examined (Fig. 3). Compared with the distribution of wtGat1p (Fig. 1B), all nuclear Gat1p_S360A_, _S361A_, Gat1p_1–375_, and Gat1p_1–375, S360A, S361A_ increased (from 9.2% to 22%, 28% and 44%, respectively) (Fig. 3). The enhancing effect of S360A and S361A on nuclear distribution was stronger than single mutations, thus further confirmed the previous results (Fig. 2B). Since the derepressing effect of Gat1p_1–375, S360A, S361A_ was the better, this metabolic engineering strategy was chosen to perform further qRT-PCR examination.

**Figure 4:**
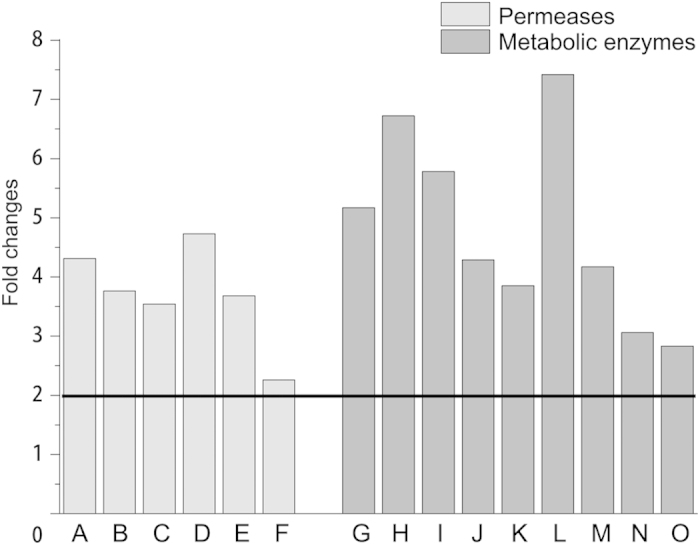
The derepressing effects of modified Gat1p on the transcriptional level of non-preferred nitrogen metabolic genes. Light and dark gray bars indicate the fold changes in the transcriptional level of non-preferred nitrogen permeases and metabolic enzymes, respectively. The strain with pYX212 was used as the control. Genes A–O represent *CAN1*, *DAL4*, *DAL5*, *DUR3*, *PUT4*, *UGA4*, *CAR1*, *DAL1*, *DAL2*, *DAL3*, *DAL7*, *DUR1,2*, *PUT1*, *UGA1*, *UGA2*, respectively. Error bars represent the standard deviation of mean of three replicates.

When the medium was supplemented with glutamine (preferred nitrogen), the transcriptional level of fifteen genes were examined. These genes are the main metabolic genes for five kinds of NCR-sensitive non-preferred nitrogen (γ-aminobutyrate, allantoine, arginine, proline and urea). Among them, six genes encode the permeases (Group one), including *CAN1*, *DAL4*, *DAL5*, *DUR3*, *PUT4*, *UGA4*. As for the other nine genes (Group two), including *CAR1*, *DAL1*, *DAL2*, *DAL3*, *DAL7*, *DUR1,2*, *PUT1*, *UGA1*, *UGA2*, they encode metabolic enzymes for relative non-preferred nitrogen. The qRT-PCR results showed that the transcriptional level of all tested genes was enhanced in the yeast (Gat1p_1–375, S360A, S361A_) when compared with the strain transformed with pYX212 (fold changes > 2), but the extent for each gene was different (Fig. 4). The derepressing effect on group two (encoding metabolic enzymes) was more significant than group one (encode permeases). As for the solo non-preferred nitrogen, based on the changing extent of metabolic genes, the order of NCR derepressing effect was as follow: urea > allantoine > arginine > proline > γ-aminobutyrate. Based on the qRT-PCR results, it is clear that new identified NLS and NLRS for Gat1p play a vital role in the regulation of NCR-sensitive genes and the modifications on Gat1p could enhance their transcriptional level.

### The utilization of non-preferred nitrogen during shaking-flask culture tests

**Figure 5:**
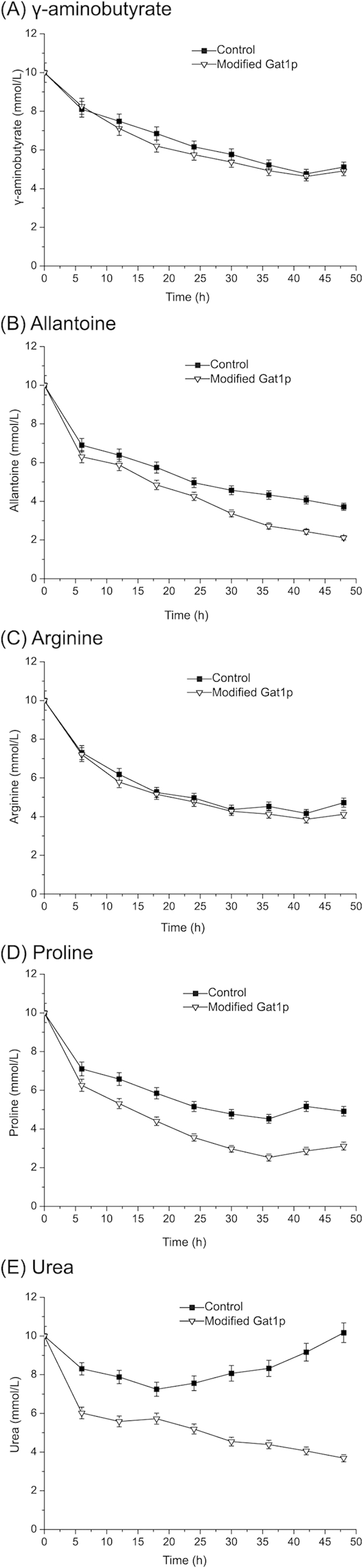
The effects of modified Gat1p on the non-preferred nitrogen utilization during shaking-flask culture tests. The strains were cultured in YNB media supplemented with non-preferred nitrogen (γ-aminobutyrate, allantoine, arginine, proline and urea) and glutamine (10 mM). The strain with empty pYX212 was used as the control. The residual non-preferred nitrogen content of the media was measured every 6 hours over the culture period. Error bars represent the standard deviation of mean of three replicates.

The qRT-PCR results confirmed the derepressing effect of Gat1p on the transcriptional level, but its effect on the utilization of non-preferred nitrogen during shaking-flask culture had not been tested. Therefore, the strains with pYX212-Gat1p_1–375, S360A, S361A_ were cultivated in YNB media with a moderate amount of non-preferred nitrogen (γ-aminobutyrate, allantoine, arginine, proline and urea) for 48 h. The strain transformed with pYX212 was used as the control. As for urea, its concentration increased from 8 h for the control. In contrast, the consumption of urea was faster in the initial stage of culture and urea did not accumulate during the 48 h culture for the genetically modified strain (Fig. 5E). Urea utilization for strain with Gat1p_1–375, S360A, S361A_ reached 63.2%, which was significantly improved compared with the control. The concentration of allantoine and proline both decreased for genetically modified strain and control in their shaking-flask culture tests, but the allantoine and proline utilization increased from 62.1% and 50.2% to 78.9% and 68.7%, respectively (Fig. 5B,D). The results of γ-aminobutyrate and arginine were different. The utilization of these two nitrogen (48.6% and 52.8%) did not significantly change compared with the control (50.7% and 58.3%) by the modification on Gat1p (Fig. 5A,C).

## Discussion

Previous studies have shown that at least four regulators (Gln3p, Gat1p, Dal80p, and Gzf3p) are involved in the NCR pathway in *S. cerevisiae*^3^. Of these, Gln3p has been intensively investigated in recent years^15^,^16^. However, these researches on Gat1p have mainly focused on the regulatory mechanism of Gat1p, and the genes that it directly regulates^6^,^17^. In this study, based on a sequence alignment of Gln3p and Gat1p, the putative NLS of Gat1p was preliminarily predicted, but no similar NLRS was identified in Gat1p. Fluorescence detection of its nuclear localization confirmed that the predicted NLS for Gat1p was correct. Without this region, Gat1p was retained in the cytoplasm under all conditions. Furthermore, a rough NLRS of Gat1p was identified when the protein was truncated. Similar to Gln3p, the NLRS of Gat1p is also located in the C-terminal region^11^.

Based on the NLS and NLRS information of Gat1p, the roles of these regions in NCR regulation were investigated. Compared with our previous work, the derepressing effects of the mutated or truncated Gat1p were much greater than those of Gln3p^12^. The urea utilization for the strain with Gat1p_1–375, S360A, S361A_ (63.2%) was higher than the strategies used on Gln3p (Gln3p_1–653, S344A, S347A, S355A_) (55.4%). This phenomenon showed that the regulation of Gln3p and Gat1p are different^18^. As the derepressing effect of NLRS truncated Gln3p was dependent on the partial disruption of interaction between Tor1p and Gln3p^11^, the effect of truncation on Gat1p indicated that the NLRS of Gat1p also have a similar capacity to interact with Tor1p and the interaction between Tor1p and Gat1p was much weaker without the rough NLRS region.

Besides the remarkably derepressing NCR effect of modified Gat1p, several noticeable results attracted our attention. Firstly, the transcriptional fold changes for the genes encoding metabolic enzymes were higher than the genes encoding permeases. This similar phenomenon was also observed in previous research^2^,^19^. It meant that the regulation of two kinds of genes are different. Apart from NCR, the expression of permeases were also controlled by posttranslational modification (ubiquitination)^20^,^21^. Secondly, the examinations of transcriptional level did not fully correspond to the shaking-flask culture tests. Compared with the utilization of urea, allantoine and proline, the transcriptional level of *CAN1*, *CAR1*, *UGA1*, *UGA2* and *UGA3* (encoding metabolic enzymes and permeases for arginine and γ-aminobutyrate) were enhanced by modified Gat1p, but the utilization of arginine and γ-aminobutyrate did not increase. This result confirms that NCR is not the only regulatory mechanism for non-preferred nitrogen metabolism^2^,^22^. Thirdly, among five non-preferred nitrogen used in this study, the urea was the only one that could be accumulated during the shaking-flask culture tests. The similar phenomenon had been appeared in our previous work^2^,^12^. In the initial stage of culture, the metabolism (strongly) and transport (slightly) of urea was repressed when preferred nitrogen (glutamine) existed^3^. As a result, a certain amount of urea was transported into cells and the intracellular concentration of urea increased. As the high level of urea was harmful for the growth of cells^2^, urea was transported into the media at the end of growth phase^23^. In the contrast, the strains with modified Gat1p could derepress the regulation of NCR, thus the concentration of urea did not increase at the end of growth phase. Similar with the effect of modified Gln3p, urea accumulation could be significantly reduced when modified Gat1p was used in the model rice wine production^12^.

Based on the results obtained in this study, the effect of NCR regulation on non-preferred nitrogen metabolism could be derepressed by weakening the phosphorylation of Gat1p. Although the NCR pathway is beneficial for the fitness of *S. cerevisiae* under different conditions, it can also result in the accumulation of some non-preferred nitrogen sources, such as urea, arginine, and proline in industrial biotechnological processes^19^,^24^. Not only this result in the waste of substrate, it can also cause food safety issues, such as the accumulation of biogenic amines^25^,^26^ and ethyl carbamate^26^, during the production of many fermented foods. The findings and strategies presented in this work could be further applied to solve these problems by the rational regulation of the NCR pathway.

## Materials and Methods

### Strains and culture conditions

*Saccharomyces cerevisiae* CEN.PK2-1C (*MATa*; *ura3-52*; *trp1-289*; *leu2-3_112*; *his3*Δ*1*; *MAL2-8*^*c*^; *SUC2*) was obtained from the European *Saccharomyces cerevisiae* Archive for Functional Analysis (EUROSCARF)^27^. The multiple-copy plasmid pYX212 was obtained from R&D systems (R&D Systems, Inc., Wiesbaden, Germany)^28^. YPD medium (10 g/L yeast extract, 20 g/L peptone, 20 g/L glucose) was used to promote the activated growth of yeast. As glutamine is one of the most preferred nitrogen sources and proline is one of classic non-preferred nitrogen sources for yeast, glutamine or proline (10 mM) was added in the YNB medium (1.6 g/liter yeast nitrogen base with no ammonium sulfate and amino acids, 20 g/liter glucose) to examine the derepressing effect of metabolic engineering on Gat1p^12^. All the strains used in this study were cultivated in 50 mL of media at 30 °C with shaking at 200 rpm. All experiments were performed in biological replicates for each sample and mean values were used for further analysis.

### Cloning and vector construction

All the primers used for vector construction are given in Table 1. The construction of pYX212–*EGFP* was described in previous work^2^. The *GAT1* gene was inserted into the *Sal* I and *Sac* I sites of pYX212–*EGFP* to construct the vectors for fluorescence localization detection. (Gly+Ala)_5_ was used as the linker between the EGFP and Gat1p^29^. In addition, the *GAT1* gene was cloned into pYX212 (digested with *Nco* I and *Sac* I). All of the constructs were verified by DNA sequence analysis. *S. cerevisiae* CEN.PK2-1C was transformed with the verified plasmids by the lithium acetate method^30^, with selection on YNB medium agar plates (adding 40 mg/L histidine, 40 mg/L tryptophan and 40 mg/L leucine)^12^.

### Mutation and truncation of Gat1p

All the primers used in mutation and truncation protocols are given in Table 1. Site-directed mutations were inserted in the phosphorylation sites using the MutanBEST Kit (Takara, Dalian, China). The correct mutation of the target genes was confirmed by DNA sequencing. The correct plasmids were transformed into *S. cerevisiae* CEN.PK2-1C for expression. Gat1p truncation inserts were also amplified from the genomic DNA of *S. cerevisiae* CEN.PK2-1C and cloned into pYX212 or pYX212–*EGFP*^12^.

### cDNA synthesis and qRT-PCR

Yeast were pre-cultured in YPD medium, and subsequently transferred into fresh YNB medium with glutamine (10 mM) and incubated at 30 °C. The samples were harvested at 12 h according to the previous conditions^12^. The steps for cDNA synthesis and qRT-PCR were the same as given in previous work^12^. All the related primers used in qRT-PCR are listed in Table 2. The wild-type *S. cerevisiae* CEN PK2 strain with empty pYX212 plasmid was used as the control. All of the fold changes of transcriptional level of non-preferred nitrogen metabolic genes were calculated as follow: ΔΔCt_sample_=(Δct_target_/Δct_reference_)/(Δct_control_/Δct_reference_). Data was normalized against the expression of the reference gene, *ACT1*^31^.

### Fluorescence microscopy analysis

Yeast strains containing fusion expression vectors for EGFP and Gat1p were grown in YNB media with glutamine or proline (10 mM) until a steady state logarithmic phase of growth (20 h). The cells were observed by fluorescence microscopy with a Nikon DXM1200C camera (Nikon, Tokyo, Japan). The digital images were obtained by the automated quantitative image software Nikon NIS-elements (Nikon, Tokyo, Japan)^32^. In order to calculate the percentage of intracellular distribution of EGFP-Gat1p truncated proteins, one hundred cells were randomly chosen and scored for different samples in digital images^32^. The standard deviation from the mean of three independent measurements for each sample was <5%. Based on the results of fluorescence microscopic analyses, the truncated proteins in the cells were classified into three categories (cytoplasmic, nuclear-cytoplasmic or nuclear).

### Shaking-flask culture tests

Yeast were pre-cultured in YPD medium, and subsequently transferred into fresh YNB medium with non-preferred nitrogen (10 mM) and glutamine (10 mM) and incubated at 30 °C for 48 h with continuous shaking at 200 rpm. Samples were taken every 6 h to measure the concentration of non-preferred nitrogen in the broth. Analyses of arginine, proline and γ-aminobutyrate were performed using the Agilent 1200 HPLC system (Palo Alto, CA, USA) and a Zorbax Eclipse AAA (4.6 × 150 mm) column^33^. The concentration of urea and allantoine were determined by the previously described methods, respectively^34^,^35^.

## Additional Information

**How to cite this article**: Zhao, X. *et al*. The modification of Gat1p in nitrogen catabolite repression to enhance non-preferred nitrogen utilization in *Saccharomyces cerevisiae. Sci. Rep.* **6**, 21603; doi: 10.1038/srep21603 (2016).

## Figures and Tables

**Table 1:** Oligonucleotides used for construction, mutation and truncation.

Gene	Forward/Reverse	Sequence (5′-3′)*
Primers for identifying potential NLS in Gat1p		
Gat1p_1–347_	F	ACGCGTCGACGATGCACGTTTTCTTTCCTTTGCTT
	R	CGAGCTCCTACAGAGGCCTTGTGACGCC
Gat1p_1–375_	F	ACGCGTCGACATGCACGTTTTCTTTCCTTTGCTT
	R	CGAGCTCCTAAGACGACGATGGAGGGGG
Gat1p_348–510_	F	ACGCGTCGACTCGTTGAAGACTGACATCATTAAGA
	R	CGAGCTCCTATAAATTCAGATTCAACCAATCC
Gat1p_376–510_	F	ACGCGTCGACCTCAATCCGGGAGCAGCAG
	R	CGAGCTCCTATAAATTCAGATTCAACCAATCCA
Primers for mutating possible phosphorylation sites in the NLS of Gat1p		
Gat1p_S348A_	F	CCTCTGGCTTTGAAGACTGACATC
	R	CCTTGTGACGCCGTGGAG
Gat1p_S348D_	F	CCTCTGGATTTGAAGACTGACATC
	R	CCTTGTGACGCCGTGGAG
Gat1p_T351A_	F	TTGAAGGCTGACATCATTAAGAAGAG
	R	CGACAGAGGCCTTGTGACG
Gat1p_T351D_	F	TTGAAGGATGACATCATTAAGAAGAG
	R	CGACAGAGGCCTTGTGACG
Gat1p_S360A_	F	CAGAGGGCTTCTACCAAGATAAAC
	R	TCTCTTCTTAATGATGTCAGTCTTC
Gat1p_S360D_	F	CAGAGGGATTCTACCAAGATAAAC
	R	TCTCTTCTTAATGATGTCAGTCTTC
Gat1p_S361A_	F	CAGAGGTCGGCTACCAAGATAAAC
	R	TCTCTTCTTAATGATGTCAGTCTTC
Gat1p_S361D_	F	CAGAGGTCGGATACCAAGATAAAC
	R	TCTCTTCTTAATGATGTCAGTCTTC
Gat1p_T362A_	F	CAGAGGTCGTCTGCTAAGATAAAC
	R	TCTCTTCTTAATGATGTCAGTCTTC
Gat1p_T362D_	F	CAGAGGTCGTCTGATAAGATAAAC
	R	TCTCTTCTTAATGATGTCAGTCTTC
Primers for expressing Gat1p and truncated Gat1p fragments		
pYX212-Gat1p	F	CATGCCATGGACGTTTTCTTTCCTTTGCTT
	R	CGAGCTCCTATAAATTCAGATTCAACCAAT
pYX212-Gat1p_1–375_	F	CATGCCATGGACGTTTTCTTTCCTTTGCTT
	R	CGAGCTCCTAAGACGACGATGGAGGGGG

^*^Restriction enzyme cleavage sites or mutation sites are underlined.

**Table 2:** Oligonucleotides for qRT-PCR.

Gene	Forward/Reverse	Sequence (5′-3′)	Product size (bp)	Mean PCR efficiency
*ACT1*	F	TTATTGATAACGGTTCTGGTATG	100	1.926
	R	CCTTGGTGTCTTGGTCTAC		
*CAN1*	F	GTGATGAAGATGAAGGAGAAG	199	1.904
	R	TGCGTGACAGAATATGCC		
*CAR1*	F	AGAGCAGATTTGGTTGGTGAAG	179	1.927
	R	CAGCGTGGGCGTCTATCC		
*DAL1*	F	CCAGACGGAGCCACCTAC	105	1.930
	R	CACTACTGAGCCTATAACACCTT		
*DAL2*	F	GTATTCTGACCAACACTTCG	175	1.922
	R	TGCCGTATCAACAATAATCTTC		
*DAL3*	F	TGGTGGCGGAGACATTGAC	200	1.931
	R	CTGTGGAAAGCAACGGAATAGG		
*DAL4*	F	GGAGACCACTTACACCAGAGG	195	1.929
	R	GGACAGATACATAGAGCCATTGC		
*DAL5*	F	ATCTCGCCCGTCTCATTTATTTG	198	1.911
	R	AACAGCATAACATACGACCATAG		
*DAL7*	F	AACCGAACAAATCAGGAAC	174	1.925
	R	CAAGTTGGAGATGAAGAGTC		
*DUR1,2*	F	GGTGTCCCTATTGCTGTTAAG	186	1.943
	R	CCGTGTGCCGACTAATCC		
*DUR3*	F	ACTGCCTGTGGGTGTTGTTG	200	1.932
	R	CGTCTACTGGATGCCTCTTGG		
*PUT1*	F	TCCGACACACTCTAACAC	154	1.941
	R	CGCAATACCGATGAATCC		
*PUT4*	F	CCTTCGCCTTCATTCTTG	154	1.930
	R	GGAACAATAACGGAGATGG		
*UGA1*	F	CAGCACAATCACCAGAAATG	199	1.908
	R	TTGGCACGGTAATAAATAAAGG		
*UGA2*	F	GTATTCTAAACCAACTCTAAACG	177	1.928
	R	GCAACATCAATCGCTTCC		
*UGA4*	F	GATGATGCCGCCACTGATG	169	1.933
	R	AAGCAACACCTGTCCAAGC		
